# Annotations

**Published:** 1889-11-09

**Authors:** 


### Annotations.

**The Case of Dr. Moon.** The trial of Dr. Moon, of Greenwich, on the charge of procuring abortion has ended in a verdict of not guilty on every count, in which the judge, Mr. Justice Stephen, expressed his concurrence. Thus ends one of the most painful medical trials of modern times, and we congratulate Dr. Moon upon his acquittal from the disgraceful charges. As the judge pointed out, Dr. Moon is a man advanced in life, and several representative men, medical and lay, gave him a character since he was a student. He has been in practice at Greenwich for many years, but was put on his trial for a crime in which he had no interest, except a paltry sum of 5s. The woman, however, went to Dr. Moon's surgery for the purpose of procuring abortion, and the case of Dr. Moon was, in which the jury acquiesced, that she carried out her object by a base deceit, and so made him the blind and innocent cause of her death. Dr. Pepper and others stated that there were a number of women who were shameful enough to practise such deception on medical men, and we ourselves have met with such cases in the course of our practice. Of course these women, by such practices, expose medical men to the danger of having money extorted from them by the persons operated upon, of exposure, or of having to answer a criminal charge. This case will give the public an inside view of some of the trials and difficulties attending medical practice, despite the utmost care and discretion on the part of practitioners generally. It was, of course, contended by the prosecution that no proper care was exercised in the case under notice or the deception could never have succeeded. All we can say is, as the result of considerable practice in similar cases, that patients of this description will stick at nothing, and by long practice they have been known to deceive the most experienced practitioners. This will be readily admitted by those who have an intimate knowledge of out-patient work.

**St. John's Hospital for the Skin.** The last service which *Truth* has rendered to the public consists in its persistent insistance upon a full, impartial, and exhaustive public investigation into the affairs of the St. John's Hospital for the Skin. Unfortunately, hospital managers know too well how evil has been the history of the St. John's Hospital, and how it has injured the cause of all the voluntary charities by its shameful mismanagement over a long period of years. In these circumstances, and on public grounds, we rejoice to be able to announce that the Princess of Wales has withdrawn from all connection with this hospital. It is further satisfactory to call to mind the fact that Mr. Savage, the honorary solicitor to the hospital, and Mr. Percy Gye, the honorary standing counsel, have both retired. As *Truth* points out, the disassociation of these two gentlemen from the Mercier faction is a circumstance of considerable significance. "Mr. Savage was something more than the honorary solicitor to the St. John's Hospital. He had, in the avowed capacity of Mr. St. Vincent Mercier's friend as well as his legal adviser, taken up the cudgels in defence of that gentleman when first the financial irregularities at the hospital were exposed in *Truth*. He had acted as solicitor for Mr. Mercier throughout the actions for libel which followed against *Truth* and against the proprietor of *Ally Sloper's Half-Holiday*. He had a better opportunity than any man living—except Mr. St. Vincent Mercier himself—of knowing how far the Lord Chief Justice was warranted in saying, as he did at the conclusion of the *Truth* case, that the demand for enquiry into Mr. St. Vincent Mercier's financial eccentricities was amply justified by that gentleman's own admissions in the witness-box. It stands to reason, therefore, that after his long connection with the hospital and his intimate association with the Merciers, knowing, moreover, as he must have known, that his withdrawal would necessarily suggest inferences most unfavourable to the Mercier interest, Mr. Savage did not sever his connection with St. John's Hospital without some very strong reason. The fact that Mr. Percy Gye thought it expedient to follow the solicitor into retirement, lends additional significance to Mr. Savage's action." That the Princess of Wales should have also retired speaks volumes for the present condition of affairs, and we repeat, as we have before urged more than once, that until a full and impartial public investigation into the management of this hospital takes place no responsible person is justified, in our opinion, in contributing to its funds. We would further support the position taken up by *Truth*, in reference to the resignation of Mr. Savage, and of Mr. Percy Gye, whose high professional attainments and personal character are widely appreciated. These gentlemen, under the circumstances of the case, are undertaking a very great responsibility if they refrain any longer from stating the precise reasons which have led them to retire from all connection with St. John's Hospital. To do less than this will be to place themselves in opposition to the best interests of the public and of the whole system of voluntary charities. We cannot believe, therefore, that they will longer withhold a frank and full statement of all the facts of the case, so far as they may be within their knowledge. The withdrawal of the Princess of Wales, which was not decided upon without the fullest consideration, and the refusal of a grant from the Hospital Sunday Fund, will convince the most impartial persons that the management of this particular hospital must either be publicly investigated, or else steps must be taken to close the institution altogether.

**Is Woman the Lesser Man?** Tennyson, in his younger days, thought that woman was the "lesser man" and in "Locksley Hall" said one or two things about moonlight and sunlight and water and wine which we are too polite to quote. Darwin held the same views, and at p. 51 of the "Origin of Species" says: "Male and female children resemble each other closely, like the young of many other animals in which the adult sexes differ widely; they likewise resemble the mature female much more than the mature male. The female, however, ultimately assumes certain distinctive characters, and in the formation of her skull is said to be intermediate between the child and the man." This agreement of the poet and the man of science on a practical question of so much importance demands a little consideration on the part of women. It is more than probable that many of them would decidedly incline to question the conclusions expressed, notwithstanding the eminence of the authorities. The present writer ventured a few evenings ago to suggest in the presence of ladies that perhaps one reason why women required more sleep than men was to be found in the more childlike and immature condition of the brain—immature, that is, as compared with a quite mature male brain. The suggestion was received with a good deal of mild derision. Nevertheless, with such excellent support as the opinions of Tennyson and Darwin, there is no need to hastily withdraw the view expressed. It is really, as we have said, a practical question of much importance. For, if the brain of the average woman be more like that of the child than that of the man, it is clear that the mental work assigned to women should not be such as is assigned to men, but should approach that which is usually given to children. On this assumption the conclusion that women are well adapted to such pursuits as medicine, law, and so on, requires reconsideration. Not only so, but Girton and Newnham may find it desirable to put forward a different programme, and one more suited to the somewhat immature condition of the female brain. Of course it cannot be expected that actual changes will be made merely on the dicta of a great poet and a famous philosopher. Practical experience will alone guide to practical changes. In the meantime, however, the opinions of two such distinguished persons, arrived at as the result of the consideration of such widely divergent classes of facts, are eminently worthy of the attention of rational persons of both sexes.

